# Tumor-Driven Inflammation Promotes Tumor Growth: A Focus on Anti-Inflammatory Cancer Treatments

**DOI:** 10.3390/diseases14080294

**Published:** 2026-08-14

**Authors:** Victor Ivanovich Seledtsov

**Affiliations:** Institute for Fundamental and Clinical Immunology, 630099 Novosibirsk, Russia; seledtsov@rambler.ru; Tel.: +7-915-2636027

**Keywords:** inflammation, tumorigenesis, pro-tumor immunity, anti-tumor immunity, anti-inflammatory treatment, immunotherapy, cancer prognosis

## Abstract

Inflammation can either encourage or suppress tumor growth, thus having a two-sided effect on cancer development. This depends on the balance between pro-tumor and anti-tumor immune responses within the tumor microenvironment (TME). Pro-tumor inflammation, driven by specific immune cells, enhances blood flow and nutrient supply to tumors, promoting the activation of dormant cancer cells (DCCs). Conversely, antitumor inflammation hinders blood flow and can force active cancer cells into a state of dormancy. Tumors actively shift this balance towards pro-tumor inflammation to create a favorable environment for growth. Therefore, anti-inflammatory therapy may be an integral part of comprehensive cancer immunotherapy. This review explores how different anti-inflammatory medications, such as glucocorticoids, non-steroidal anti-inflammatory drugs (NSAIDs), antihistamines, anti-leukotrienes, statins, drugs that block pro-inflammatory cytokines, agents that inhibit oxidative phosphorylation, antioxidant vitamins, anti-angiogenic drugs, and low-dose chemotherapy, can be used to combat cancer. Granulocyte counts and erythrocyte sedimentation rate (ESR) can be used to assess inflammation levels and the effectiveness of anti-inflammatory treatments. We advocate for a paradigm shift in cancer treatment, moving away from aggressive tumor destruction, which triggers uncontrolled tumor regeneration, toward long-term immunological control of tumor growth while preserving the patient’s overall health.

## 1. Introduction

The immune system is crucial for maintaining the integrity and function of the body by defending against pathogens and regulating cell growth for repair. Typically, the immune system perceives tumors as healthy tissues that require repair [1]. The fundamental innate and adaptive immune mechanisms driving tumor progression largely mirror those involved in regenerative cell activity in the normal tissues. These mechanisms are tightly regulated in acute wounds but become considerably unbalanced in tumors [2].

The destruction of tumor cells can paradoxically stimulate the generation of more tumor cells because of the tumors’ abnormal regenerative capacity. The adaptive capabilities of tumor cells can decrease their susceptibility to cytotoxic drugs during treatment, often preventing complete tumor eradication [3]. Moreover, the detrimental side effects of cytotoxic treatments on healthy cells and tissues significantly restrict their clinical applications. Thus, gentler strategies that directly or indirectly inhibit tumor growth may prove more effective in the long term than methods that focus solely on tumor destruction. As a tumor expands, it leverages regenerative mechanisms and induces inflammation to promote its growth. Anti-inflammatory therapy aims to counter inflammation, establishing a metabolic and immunological microenvironment around the tumor that impedes its progression.

This review addresses three key questions: (1) What evidence supports the role of inflammation in regulating tumor dormancy and progression? (2) Which anti-inflammatory agents demonstrate the strongest preclinical and clinical evidence of anticancer effects? (3) How can anti-inflammatory therapy be optimally integrated with immunotherapy across different cancer stages?”

## 2. Tumor-Driven Inflammation

Chronic low-grade inflammation is now understood to be a crucial element in tumor initiation and development. Increased cell proliferation in response to chronic tissue damage increases the sensitivity of cells to the action of mutagenic factors. Immune cell-derived reactive oxygen species (ROS) trigger cancer through a complex process that begins with direct DNA damage and progresses to changes in cell signaling. The inflammatory tumor microenvironment (TME), enriched with cytokines and chemokines, fosters angiogenesis and genomic instability, but also downstreams signaling pathways, including NF-κB, STAT3, and COX-2/PGE2, bridge inflammation and oncogenesis [4]. Mounting evidence convincingly demonstrates the crucial role of inflammation in cancer development. Studies have shown that aspirin and other NSAIDs can lower the incidence of various cancers, including those of the breast, bile duct, colon, endometrium, esophagus, stomach, liver, lungs, ovaries, prostate, pancreas, and digestive system [5].

We previously showed that tumor cells resistant to mutual contact-dependent interactions remain sensitive to contact-dependent growth inhibition by normal cells such as immune cells. Tumor cells, located in a dense environment of normal cells, can probably remain in an inactive (dormant) state for a long time without developing disease. To realize its growth potential, a tumor must first reach a critical mass and form an actively growing center [6,7]. However, in the absence of additional oxygen, nutrients and growth factors, tumor cells can continue to remain dormant for long periods of time. Tumor dormancy is a dynamic and reversible state in which cancer cells enter a period of cell cycle arrest and remain in a non-proliferative or slowly cycling state for extended periods without lesion formation. Cancer cells can enter a dormant state at any stage of cancer development. Dormant cancer cells (DCCs) resemble and occasionally coincide with quiescent or slow-proliferating cancer stem cells [8]. Dormancy arises from a specific epigenetic signature rather than gene mutations. The phenomenon of tumor dormancy and its “awakening” explains why cancers can recur years or even decades after seemingly successful treatment [9,10].

Hypoxia is a critical factor in inducing and maintaining tumor dormancy [11]. Under hypoxic conditions, cells activate adaptive stress programs primarily governed by hypoxia-inducible factor 1-alpha (HIF-1α), which drives transcriptional changes that promote survival and entry into a quiescent state. DCCs often persist in poorly vascularized, oxygen-deprived niches, a phenomenon sometimes referred to as ‘angiogenic dormancy.’ Cancer cells under hypoxic conditions upregulate dormancy markers, such as transforming growth factor--β (TGF-β), NR2F1, and p27 [12]. Nutritional deprivation is another key factor that facilitates tumor dormancy. DCCs undergo metabolic reprogramming to adapt to limited nutrients and stress [13]. Notably, AMPK activation, a key energy sensor, shifts cancer cells from anabolic growth to catabolic metabolism to support survival under stress [8].

The TME is crucial in controlling whether cancer cells remain dormant or awaken, both at the primary tumor site and secondary locations. Oxygen supply restoration, often driven by angiogenesis, resolves hypoxic stress, leading to the reactivation of DCCs through the downregulation of hypoxia-responsive genes and the reactivation of oxidative metabolism [14].

The destruction of both tumor and normal cells, whether naturally occurring or induced, releases significant amounts of damage-associated molecular patterns (DAMPs) into the TME. These DAMPs act as intrinsic danger signals, promoting regenerative inflammation that favors tumor growth [15]. This inflammation improves blood flow to the tumor and promotes DCC activation. In turn, active tumor cells recruit pro-tumor immune cells (N2 neutrophils, M2 macrophages, myeloid-derived suppressor cells, Th2 cells, and regulatory T and B cells) into the TME and activate immune checkpoints [3]. Cytokines and chemokines released by recruited immune cells sustain pro-tumor inflammation, thereby promoting tumor cell proliferation, survival, and metastasis [16]. Conversely, the immune system can fight tumor progression by initiating inflammation that suppresses tumor growth. This inflammation is orchestrated by specific antitumor immune cells, namely N1 neutrophils, M1 macrophages, NK cells, NKT cells, γδ T cells, Th1 cells, and CD8+ cytotoxic lymphocytes. It inhibits anti-angiogenesis and impairs blood supply to the tumor [3,17]. In this inflamed environment, T cells eliminate active tumor cells that display MHC molecules bound to neoantigenic peptides on their exterior. While CD8+ T and NK cells, with help from CD4+ cells, use interferon-gamma to induce dormancy in cancer cells, different signals—tumor necrosis factor α (TNF-α), interleukin-6 (IL-6), IL-8, IL-17 and CXCL1 from CD4+ T cells—act as “wake-up” calls to reactivate them [18,19,20].

**Figure 1:**
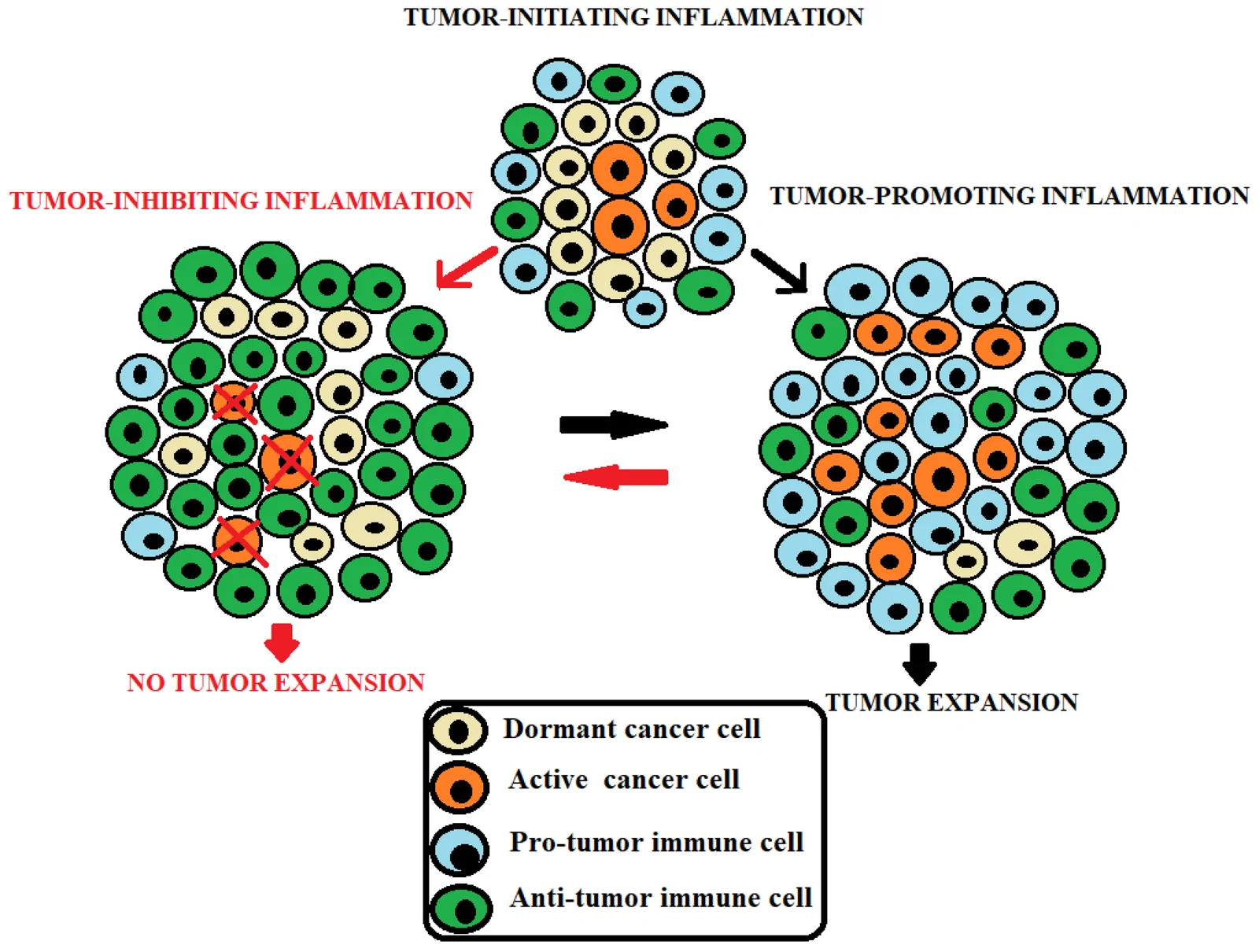
Conceptual presentation of inflammatory regulation of tumor growth. Pro-tumor inflammation activates dormant cancer cells (DCCs) and promotes tumor protection, whereas anti-tumor inflammation drives active tumor cells into a DCC state and facilitates tumor destruction. The overall tumor growth rate is determined by the balance between these opposing inflammatory forces in the TME.

As illustrated in Figure 1, a “cold” tumor’s transition to a “hot” one can cause either enhanced or reduced tumor growth, contingent upon the prevailing pro- or anti-tumor inflammatory activity in the TME. The inflammatory balance within the TME ultimately dictates the overall tumor growth. A single tumor can simultaneously harbor regions with various cell growth activity, which dynamically shift in size and location [3].

The role of different types of immune cells in regulating tumor growth may be ambiguous. For example, cytolytic M1 macrophages can awaken DCCs through the secretion of pro-inflammatory cytokines such as TNF-α and IL-6, promoting tumor expansion; conversely, immunosuppressive regulatory T cells, by producing TGF-β and suppressing the immune cell secretion of pro-inflammatory cytokines in the TME, can maintain the tumor’s dormant state and thus inhibit tumor expansion [21]. Considering the immune-mediated regulation of tumor growth described here, the roles of different immune cells in counteracting tumor expansion may be re-evaluated, and the immunotherapeutic concept significantly revised.

A key biological feature of DCCs is their resistance to chemoradiotherapy, which is primarily attributed to their lack of cell proliferation. Recent preclinical studies have proposed two broad strategies for targeting DCCs: (1) activating DCCs to sensitize them to conventional therapies and (2) maintaining their quiescent state to prevent proliferation and metastasis [8]. Given the pathologically high regenerative potential of tumors, the second strategy appears to be preferable. This approach offers the possibility of long-term tumor growth control without systemic toxicity to the host.

As the tumor mass increases, its inflammatory and pro-tumorigenic effects on neoangiogenesis and regenerative immunity intensify, rendering tumor growth increasingly uncontrollable. Therefore, anti-inflammatory therapy could be administered throughout all stages of the disease and intensified as the cancer progresses. This therapeutic approach aims to (i) restrict blood flow to the tumor, prompting active tumor cells to become dormant, and (ii) bolster the effectiveness of immunotherapies against the tumor, such as antibody-based immunostimulation, drug-mediated immunomodulation, adoptive cell immunotherapy, donor leukocyte transfusion, and cancer vaccination [22].

## 3. Anti-Inflammatory Drugs as Tumor Growth Inhibitors

### 3.1. Glucocorticoids

Glucocorticoids (GCs) are a cornerstone of cancer therapy, used primarily for their potent anti-inflammatory and immunosuppressive effects. GCs are commonly used to manage adverse events associated with immunotherapy (irAEs) and are regularly employed to reduce brain swelling, control nausea and vomiting, alleviate spinal cord compression, and avert hypersensitivity responses to medications [23].

GCs inhibit angiogenesis by binding to the glucocorticoid receptor, which subsequently regulates the transcription of target genes. The antiangiogenic properties of GCs are correlated with diminished tumor vasculature and reduced tumor growth, as shown in multiple in vivo studies [24,25,26]. Moreover, GCs exert direct cytotoxic effects on hematologic malignancies, including lymphomas, acute lymphoblastic leukemia, and multiple myeloma, by triggering apoptosis [23,26]. They are capable of inducing dormancy in solid tumor cells, such as those in lung cancer [27]. By altering the TME, GCs may either enforce quiescence or, under specific immunosuppressive conditions, contribute to the “awakening” of DCCs [28,29]. The challenge lies in weighing the beneficial anti-inflammatory effects of GCs against their detrimental immunosuppressive effects, which hinder the efficacy of immunotherapy [26,27]. According to published data [23], the use of GCs for severe irAEs in a focused, short-term manner appears to have minimal adverse effects on immunity and treatment efficacy. Future strategies should aim to achieve the necessary anti-inflammatory effects without causing significant immunosuppression.

### 3.2. Nonsteroidal Anti-Inflammatory Drugs (NSAIDs)

Chronic inflammation driven by cyclooxygenase-2 (COX-2) and prostaglandin pathways promotes tumorigenesis. COX-2 expression is upregulated in multiple malignancies, including colon and gastric carcinomas [4]. Prostaglandins (PGEs) are active participants in nearly every stage of cancer, from initiation to metastasis. The primary driver is PGE2, which signals through a set of G-protein-coupled receptors known as EP1, EP2, EP3, and EP4 [4]. PGE2 directly interacts with cancer cells to promote their proliferation and survival, often through the activation of Wnt signaling [30]. Furthermore, one of the most critical roles of PGE2 in cancer is its ability to stimulate angiogenesis and suppress immune reactivity within the TME [31]. They also participate in various cellular processes by modulating major signaling pathways, including the NF-kB, WNT, MAPK, and mTOR/PI3K/AKT pathways [32]. These data have sparked growing interest in NSAIDs, such as aspirin, and COX-2 inhibitors as potential anti-cancer agents. NSAIDs effectively reduce PGE concentrations by inhibiting the activity of COX. To date, over 100 NSAIDs have been studied, many of which have been clinically approved for use. Owing to their potent anti-inflammatory, analgesic, and antipyretic activities, they are used worldwide to treat cardiovascular events, rheumatic immune diseases, and other pain-related conditions [33].

Several preclinical and clinical studies have found that NSAIDs, especially aspirin, can reduce the incidence of various cancers by decreasing the levels of inflammatory mediators around cancer cells [4]. Furthermore, NSAID use, both pre- and post-diagnosis, is associated with a significantly reduced risk of distant metastasis in cancers such as prostate, breast, and colorectal cancer [34,35]. In contrast to GCs, NSAIDs lack significant immunosuppressive properties, allowing their safe administration with immunostimulating medications.

Table 1 displays clinical data that suggest NSAIDs might prolong survival in a notable number of cancer patients. However, these findings are not definitive and remain inconclusive. While some extensive studies and meta-analyses point to a benefit for certain patient populations, other research indicates no such effect. It is crucial to note that NSAIDs are presently employed in standard medical practice to manage complications, not as primary cancer treatments. Moreover, cytotoxic chemotherapy and NSAIDs function differently: chemotherapy directly targets and destroys fast-growing cells, while NSAIDs reduce cytotoxic drug delivery to the tumor and induce a dormant state in tumor cells, which may enhance their resistance to chemotherapy. Therefore, using these agents together may not be theoretically advisable, and separating their administration could be beneficial. Conversely, NSAIDs and anti-hormonal agents share similar mechanisms of antitumor action. Consequently, their combined effect on tumors could be additive, making their concurrent use seem reasonable. Considerable further scientific and clinical investigation is necessary to determine the optimal use of NSAIDs within comprehensive cancer therapy.

### 3.3. Anti-Histamines

Mast cells are the primary source of histamine, which is widely recognized as a proangiogenic factor that promotes the formation of new blood vessels [49,50]. Elevated histamine levels have been detected in various human malignancies, including melanoma, colon cancer, and breast cancer. Tumors expressing H1, H2, H3, and H4 receptors have been shown to establish a TME conducive to their progression [51]. Histamine can stimulate tumor cell proliferation through the H1 receptor (H1R) while simultaneously suppressing the immune system via the H2 receptor (H2R). The upregulation of H1R and H2R in cancer cells is correlated with poor prognosis [52].

Antihistamines generally exhibit antiangiogenic properties, primarily by neutralizing proangiogenic signals mediated by histamine [53]. Recent findings indicate that antihistamines, when combined with immunotherapy, significantly prolong both overall and progression-free survival in patients with cancer [52,54]. Cationic antihistamines, such as desloratadine, cyproheptadine, ebastine, loratadine, and astemizole, are associated with better clinical outcomes than non-cationic amphiphilic antihistamines [51]. Owing to their unique molecular structures, these cationic drugs accumulate rapidly inside lysosomes, raising lysosomal pH and promoting hydrogen efflux, which ultimately induces cancer cell apoptosis [55]. Ongoing research emphasizes new-generation antihistamines, which feature a substantially reduced risk of adverse effects, thereby increasing their viability as targeted anti-cancer therapies [52].

Clinical data in Table 2 suggest that both H1 and H2 antihistamines possess antitumor potential and could be incorporated into comprehensive cancer treatment. Antihistamines clearly warrant broader application in oncological practice already.

### 3.4. Anti-Leukotrienes

Granulocytes are the primary producers of leukotrienes (LTs). These lipid mediators are synthesized by the enzyme 5-lipoxygenase (5-LO) and signal through specific G protein-coupled receptors, such as CysLT_1_R and CysLT_2_R. Along with histamine, LTs are potent drivers of infiltrative inflammatory responses. They promote angiogenesis and vascular permeability, facilitating tumor expansion and cancer cell spread [60,61]. Furthermore, the overexpression of 5-LO has been documented in primary tumor cells and cancer cell lines. LTs activate anti-apoptotic signaling pathways and enhance cancer cell proliferation. In contrast, preventing 5-LO activity hinders tumor cell proliferation by triggering cell cycle arrest and programmed cell death [61,62]. In medulloblastoma, LT synthesis is critical for cell proliferation, and genetically or pharmacologically blocking this pathway dramatically inhibits tumor growth [63].

A large retrospective cohort study of over 550,000 U.S. veterans with asthma demonstrated that the use of leukotriene inhibitors, such as montelukast, was associated with a 17% to 22% reduction in lung cancer risk [64]. However, it remains unclear whether the overall antitumor effect is primarily driven by the systemic anti-inflammatory properties of LTs or by their direct anti-proliferative actions on tumorigenic cells. Consequently, current research heavily emphasizes drug repositioning and investigates the potential anti-cancer properties of established, safe, anti-allergic medications, such as montelukast and zileuton.

### 3.5. Inhibitors of Pro-Inflammatory Cytokines

Pro-inflammatory cytokines, such as IL-1β, TNF-α, and IL-6, are known to encourage tumor development, immune system evasion, and metastasis [16]. The CANTOS trial, initially aimed at preventing cardiovascular diseases, demonstrated that canakinumab, a monoclonal antibody targeting IL-1β, substantially decreased the incidence and mortality associated with lung cancer. MABp1, a monoclonal antibody targeting IL-1α, has been investigated in colorectal cancer, demonstrating possible advantages in stabilizing the disease. Limited success has been observed when infliximab (an anti-TNF-α agent) and etanercept (a TNF receptor blocker) are used in patients with advanced cancer. Nevertheless, TNF blockade is effective in managing immunotherapy-related adverse events, especially colitis triggered by immune checkpoint inhibitors [16,65]. Testing of anti-IL-6 antibodies, such as tocilizumab, clazakizumab, and siltuximab, has been conducted in individuals with prostate cancer, lung cancer, and multiple myeloma. The observed clinical outcomes have been minimal, suggesting restricted effectiveness when used alone [16]. Because cytokine inhibitors profoundly affect immune processes, their use in combination with active immunotherapy presents a significant challenge.

### 3.6. Oxidative Phosphorylation Inhibitors

Tumor growth is highly dependent on mitochondrial energy. Metformin, carboxyamidotriazole, and other drugs that suppress oxidative phosphorylation have demonstrated antitumor and anti-inflammatory properties in several experimental settings. Metformin, a synthetic biguanide widely used to treat type 2 diabetes, suppresses complex I of the mitochondrial respiratory chain complex. Metformin reduces the expression of pro-inflammatory molecules, such as NF-κB and TNF-α, thereby mitigating the cellular “immunoinflammatory profile” [66]. Furthermore, it can reduce the influx of regulatory T cells and myeloid-derived suppressor cells into the TME, favoring the development of antitumor immune responses. In a mouse model of breast cancer, metformin administration has been shown to delay carcinoma onset and progression while activating natural killer T cells and T lymphocytes [67,68]

Carboxyamidotriazole, an inhibitor of mitochondrial complex I, suppresses the NF-κB and MAPK pathways, thereby mitigating colitis and colitis-associated carcinogenesis. This effect is linked to metabolic reprogramming in macrophages [69,70]. Notably, early phase oncology clinical trials have shown that carboxyamidotriazole is manageable, with the most frequent side effects being gastrointestinal in nature and typically mild to moderate [71].

The novel oxidative phosphorylation inhibitor IACS-10759 has demonstrated efficacy against chemoresistant triple-negative breast cancer. However, clinical trials of IACS-10759 were halted because of its toxicity. More broadly, the narrow therapeutic window of newly synthesized low-molecular-weight oxidative phosphorylation inhibitors remains a significant limitation for their clinical application [72,73].

### 3.7. Statins

Statin drugs (statins) are potent inhibitors of hydroxymethylglutaryl-coenzyme A (HMG-CoA) reductase. They are widely used in medicine as lipid-lowering agents. In addition to reducing cholesterol levels, statins have anti-inflammatory properties and can inhibit angiogenesis by suppressing endothelial proliferation [74]. The efficacy of statins against colon, lung, breast, brain, muscle, and hematological cancers has been demonstrated in vitro and in vivo. However, statins have the potential to trigger epithelial–mesenchymal transition (EMT), possibly advancing tumor growth [74]. Furthermore, certain data suggest a potential link between them and an elevated risk of particular cancers, including melanoma, non-melanoma skin cancer, and prostate cancer [75]. Consequently, the findings remain inconclusive regarding the association between statin use and clinical outcomes in patients with cancer.

### 3.8. Anti-Oxidant Vitamins

Various sources confirm that most patients with cancer use vitamin supplements. It has been reported that vitamins A, C, D, and E exhibit anti-inflammatory and antitumor effects [22,76]. The anti-inflammatory effects of these vitamins are primarily associated with their antioxidant properties, which reduce ROS levels in the body. However, the role of antioxidant therapy in cancer treatment is complex and marked by significant controversy. While some studies suggest potential benefits of ROS inhibition, others indicate that antioxidants could potentially worsen outcomes [77]. This ambiguity arises because ROS play a dual role in tumor biology. On the one hand, ROS exhibit pro-inflammatory activity and can suppress T cell reactivity [78,79]. In contrast, they exhibit direct cytotoxicity and mediate neutrophil and macrophage cytotoxic activity [17,22,78]. Consequently, antioxidants can influence the body’s antitumor defense mechanisms in multiple and often opposing ways.

### 3.9. Anti-Angiogenic Drugs

Anti-angiogenic drugs (Bevacizumab, Aflibercept, Ramucirumab, Axitinib, Nintedanib, Vandetanib, Pazopanib, Regorafenib, Sorafenib, and Sunitinib) affect inflammation and tumor growth through complex mechanisms far beyond merely restricting a tumor’s blood supply. They normalize chaotic tumor vasculature, mitigate the immunosuppressive effects of specific growth factors, and reprogram the tumor microenvironment to be more hostile to cancer [80,81,82]. Currently, anti-angiogenic therapies are typically combined with chemotherapy to treat advanced tumors. Although evidence indicates that these drugs can be effectively combined with anti-PD-1 immunotherapy [83,84] and cancer vaccination [85], the potential for synergistic combinations of angiogenesis inhibitors with broader immunomodulatory interventions remains unexplored.

### 3.10. Low-Dose Cytotoxic Chemotherapy Drugs

Metronomic chemotherapy involves the frequent and regular administration of low-dose cytotoxic drugs. Common metronomic regimens utilize agents such as vincristine, capecitabine, cyclophosphamide, methotrexate, and fluorouracil. This scheduling strategy reduces pro-tumor inflammation and fosters niches in lymphoid tissues, aiding the growth of anti-tumor immune cells [3,86]. Furthermore, chemotherapy drugs delivered at sub-cytotoxic doses exhibit anti-angiogenic activity [87]. They can enhance T cell infiltration and cytotoxic activity in the TME while simultaneously depleting immunosuppressive cells [88]. Low-dose chemotherapy can support immunotherapy by optimizing tumor growth control rather than attempting complete tumor cell destruction [3].

## 4. Laboratory Control of Inflammation

As established above, the inflammatory activity of the immune system determines the tumor trajectory. Therefore, blood-based inflammatory markers provide valuable insights into cancer progression or regression. However, standard blood tests cannot distinguish between pro-tumor and anti-tumor inflammation. Inflammation caused by various stimuli is largely based on common mechanisms; therefore, the identification of inflammatory markers alone is unlikely to clearly distinguish between pro- and anti-tumor inflammation. However, the published data [3,22] clearly indicate that an actively growing tumor is a powerful inflammatory stimulus and makes the most significant contribution to the increase in inflammatory markers. Consequently, high blood neutrophil counts and erythrocyte sedimentation rates (ESR) in cancer patients usually indicate significant inflammation, tumor progression, and poor prognosis [3,22,89,90]. Thus, a rise in these two blood parameters serves as a clear indication to initiate or intensify anti-inflammatory treatments, and monitoring their dynamics during treatment can objectively reflect the effectiveness of these interventions.

## 5. Conclusions

Inflammation drives tumorigenesis at all stages of development. In advanced malignancies, pro-tumorigenic inflammation consistently outweighs anti-tumor immunity, generating a metabolic and immunological microenvironment conducive to tumor progression. This inflammatory state facilitates DCC activation and suppresses antitumor immune responses. Therefore, combination cancer therapies should incorporate anti-inflammatory strategies throughout the treatment continuum. The concept of maintaining tumor dormancy prompts a reconsideration of standard systemic approaches to treating malignant diseases In reality, cytotoxic chemotherapy is generally unable to eradicate a tumor entirely from the body. It damages healthy tissues and the immune system and stimulates pathological tumor regeneration. Therefore, it seems most appropriate, after surgically removing the primary tumor, to immediately transition to immunotherapy combined with anti-inflammatory treatment. In cases of advanced disease, it seems advisable to conduct only a short course of high-dose cytotoxic chemotherapy to reduce the overall tumor mass, followed by a transition to long-term immunotherapy plus anti-inflammatory therapy. We hypothesize that the systematic reduction in pro-tumor inflammation through multi-agent anti-inflammatory therapy will extend progression-free survival and overall survival by maintaining a higher proportion of tumor cells in a dormant state. Prospective clinical trials are necessary to test this hypothesis, including the assessment of defined biomarker endpoints (like granulocyte counts and ESR) and imaging-based evaluations of tumor dormancy.

## Figures and Tables

**Table 1:** NSAIDs in cancer therapy (clinical studies).

Medication	Study Design	Outcome	Reference
NSAIDs	In this retrospective study, 3634 patients with non-small-cell lung cancer (NSCLC) who received ICI treatment were assessed for overall survival (OS). Of these, 2336 (64.3%) had also used NSAIDs.	NSCLC patients who take NSAIDs concurrently with other therapies tend to live longer overall.	[36]
NSAIDs	A total of 127 NSCLC patients were retrospectively assessed, among whom 36 (28.3%) received NSAIDs.	NSAID users had significantly worse objective response rates (ORR) and progression-free survival (PFS) with ICI therapy compared to non-users.	[37]
Aspirin	This study was a double-blind, randomized, placebo-controlled trial that included 1103 patients. These patients had stage I, II, or III rectal cancer, or stage II or III colon cancer, and they all had somatic alterations in PI3K pathway genes. The patients were assigned in a 1:1 ratio to receive 160 mg of aspirin or matched placebo once daily for 3 years.	Colorectal cancer recurrence was significantly less common in patients with PIK3 mutations who took aspirin compared to those who took a placebo.	[38]
Indomethacin	A phase II study involved 32 patients with advanced renal cell carcinoma to investigate how well chronic indomethacin and intermittent IL-2 therapy worked clinically. Patients were given oral indomethacin and ranitidine at least a week before starting IL-2 therapy.	In renal cell carcinoma, the regimen with indomethacin shows a response rate that is comparable to other high-dose IL-2 approaches.	[39]
Celecoxib	The Randomized European Celecoxib Trial (REACT) was a phase 3, randomized, double-blind study involving 2639 breast cancer patients. Conducted across 160 centers in the UK and Germany, the trial compared two years of adjuvant celecoxib against a placebo. Eligible patients had undergone complete resection of their breast cancer and received local and systemic therapy as per standard practice. Participants were randomized in a 2:1 ratio to receive either celecoxib or a placebo.	Celecoxib’s adjuvant treatment for ERBB2-negative breast cancer did not demonstrate a DFS benefit over placebo for two years.	[40]
Celecoxib	Twenty-nine patients diagnosed with NSCLC, stages IB to IIIA, received two preoperative cycles of paclitaxel and carboplatin along with daily celecoxib before undergoing surgical removal.	In comparison with historically reported response rates, these data suggest that the addition of a selective COX-2 inhibitor may enhance the response to preoperative paclitaxel and carboplatin in patients with NSCLC.	[41]
Celecoxib	In a randomized phase II study, postmenopausal women with hormone-sensitive breast cancer and measurable disease, who experienced progression after tamoxifen treatment, were enrolled. Participants were then randomly assigned to one of two treatment arms: exemestane 25 mg daily (n = 55) or a combination of exemestane 25 mg daily and celecoxib 400 mg twice daily (n = 56).	The duration of clinical benefit was longer in the combination group (median, 96.6 weeks) compared to the exemestane alone group (median, 49.1 weeks). The addition of celecoxib did not alter the tolerability profile of exemestane alone.	[42]
Celecoxib	Exemestane 25 mg daily was administered to patients with postmenopausal metastatic breast cancer, along with either celecoxib 400 mg twice daily or a placebo. The trial was halted early, with 157 of the planned 342 participants enrolled, due to cardiovascular toxicity observed in other studies involving celecoxib.	Celecoxib appears to help reverse endocrine resistance in women with breast cancer.	[43]
Celecoxib	Twenty two postmenopausal women with estrogen receptor and/or progesterone positive stages II–III breast cancers received 8 weeks of exemestane 25 mg daily, followed by 8 weeks of exemestane 25 mg daily and celecoxib 400 mg twice daily.	The addition of celecoxib to exemestane was tolerated by the majority of women and anti-tumor response was observed.	[44]
Celecoxib	Twenty five patients with locally advanced or metastatic pancreatic cancer have been enrolled at 3 centers. The treatment consisted of intravenous gemcitabine 1000 mg/m weekly × 7 weeks and concurrent daily oral celecoxib 400 mg orally twice a day.	The addition of celecoxib to gemcitabine therapy did not demonstrate significant improvement in measured clinical outcomes in patients with advanced pancreatic cancer.	[45]
Celecoxib	Cytokine-naive metastatic renal cell carcinoma patients (n = 17) with tumors expressing ≥10% maximal COX-2 staining by immunohistochemistry received IFN-α 5 million units daily and celecoxib 400 mg orally twice daily in an open-label, single-arm phase II trial.	Celecoxib plus IFN-α in renal cell carcinoma (RCC) patients with maximally staining COX-2 tumors does not significantly enhance overall response rates over IFN monotherapy.	[46]
Celecoxib	Forty five ovarian cancer patients were administered oral celecoxib (400 mg/day) in combination with intravenous carboplatin (AUC5, q28).	Celecoxib combined with carboplatin showed promising activity and it is well tolerated in heavily treated recurrent ovarian cancer patients.	[47]
Celecoxib	In this randomized controlled study, 54 patients with metastatic colorectal cancer were randomized into 2 groups; the control group (n = 28) which received 6 cycles of folinic acid, fluorouracil and irinotecan (FOLFIRI) regimen (5-flourouracil, leucovorin, irinotecan), and the celecoxib group (n = 26) which received 6 cycles of FOLFIRI regimen plus celecoxib 200 mg twice daily. The study duration was 3 months.	Celecoxib/FOLFIRI arm showed significantly higher PFS and one-year OS when compared to FOLFIRI arm.	[48]

**Table 2:** Antihistamines in cancer therapy (clinical studies).

Medication	Study Design	Outcome	Reference
Antihistamines	Patients with locally advanced or metastatic urothelial cancer were treated with Atezolizumab. Among these patients (n = 896) individuals were identified who did and did not receive antihistamines.	Concomitant antihistamines administration was associated with improved OS, CSS, and PFS in patients receiving atezolizumab for urothelial cancer.	[56]
H1-antihistamines	The current study involved 981 individuals diagnosed with esophageal squamous cell carcinoma who underwent standard chemoradiotherapy. Out of these, 309 were placed in the non-AH1 group and 672 in the AH1 group.	H1-antihistamine use during chemoradiotherapy was found to be associated with improved OS.	[57]
H1-antihistamines	Six retrospective cohort studies, involving 2166 patients (417 antihistamine users and 1749 non-users), were included. These studies primarily focused on lung cancer (34.5%) and melanoma (13%). All patients received ICIs.	The findings indicate that H1 receptor blockade may potentiate the therapeutic efficacy of ICIs, resulting in better survival and delayed disease progression.	[58]
H2-antihistamines	Initially, 181 articles were identified; after screening and applying inclusion criteria, six studies were included (three for colorectal cancer and three for gastric cancer).	Current evidence suggests a potential survival benefit of H2-antihystamins in colorectal cancer, but insufficient for gastric cancer.	[59]

## Data Availability

Data sharing requires no permission.
